# Editorial: Advances in neuroanatomy

**DOI:** 10.3389/fmed.2024.1481303

**Published:** 2024-10-15

**Authors:** Mauro Palmieri, Alexander I. Evins, Alessandro Pesce, Alessandro Frati, Antonio Bernardo

**Affiliations:** ^1^Neurosurgery Division, Human Neurosciences Department, University of Rome “Sapienza”, Rome, Italy; ^2^Department of Neurological Surgery, Weill Cornell Medicine, Cornell University, New York, NY, United States; ^3^Department of Neurosurgery, Policlinico “Tor Vergata”, University of Rome “Tor Vergata”, Rome, Italy; ^4^IRCCS-“Neuromed”, Pozzilli, Italy

**Keywords:** anatomy, skull base, neurosurgery, neurology, neuroimaging studies, neuromolecular anatomy, spine

Knowledge of the anatomy of the CNS represents a cornerstone for any clinical advance in neuroscience. Advances in neuroanatomy regard different fields: the application of new technologies and more precise diagnostic instruments, improvements in surgery, with the research on tailored and less invasive approaches. Furthermore, in the last decades, research has been focusing on molecular and microscopic features of both the brain and spine, to get more accurate information regarding also the physiopathology of CNS diseases. Electronic microscopy allows us to study the ultrastructural features of the nervous cell structures, allowing us to explore and better understand the modifications in the presence of different diseases, like tumors, and vascular and degenerative lesions.

Several features regarding neuroanatomy interest both experimental and clinical branches and that involve neurosurgeons, neuroanatomists, neurologists, neuroradiologists, and neuropathologists. The ultimate goal of this Research Topic is to describe and understand how to improve patient care, from early diagnosis of CNS conditions to follow-up. Finding less invasive techniques to treat patients is a current trend topic in every discipline of medicine, so the idea of proposing a Research Topic on a such wide field as neuroanatomy was born from the need to make the treatment of CNS diseases more effective, safe, and comfortable for patients. An understanding of neuroanatomy is essential for developing precision medicine approaches that target specific neural circuits and their associated disorders. There are still many things to clarify regarding neuroanatomy and the development of new techniques and technologies could lead to interesting results soon.

Wei et al. in their article entitled “*The efficacy and adverse events of bevacizumab combined with temozolomide in the treatment of glioma: A systemic review and meta-analysis of randomized controlled trials*” propose a meta-analysis on a trending topic in neurosurgery: adjuvant treatments in glioma surgery. More specifically, they report a study conducted on eight trials comprehending 3,039 participants, in which the combined use of Bevacizumab and Temozolomide (TMZ) compared to TMZ alone could significantly improve PFS, OS, and complete remission rate. However, a total of six studies reported related adverse events, mainly including thrombocytopenia, neutropenia, leukopenia, anemia, and fatigue. Therefore, subsequent studies should continue to conduct larger, multi-center RCTs to confirm the findings and explore in depth how to minimize and manage adverse events effectively.

Moving from oncology to functional disorders, Sun et al. in their article entitled “*The white matter characteristic of the genu of corpus callosum coupled with pain intensity and negative emotion scores in patients with trigeminal neuralgia: A multivariate analysis*” present an interesting original article regarding the correlation between anatomical variability and a specific condition: trigeminal neuralgia. The Authors presented the data regarding the white matter structure of 46 patients affected by chronic trigeminal neuralgia and compared them with 35 healthy controls. The analysis has been performed on T1 weighted magnetic resonance imaging and diffusion tensor imaging and the results of the multivariate analysis highlighted an interesting result: patients affected by chronic trigeminal neuralgia present major integrity of some white matter structures, particularly the genu of the corpus callosum, suggesting that this pathway is strongly involved in pain processing.

Moving to the surgical importance of anatomical landmarks in brain surgery, Fava et al., in their article entitled “*The anterior sylvian point as a reliable landmark for anterior temporal lobectomy in mesial temporal lobe epilepsy: technical note, case series and cadaveric dissection*” present the result of original research regarding the identification of the anterior sylvian point as the first step of anterior temporal lobectomy. In this study, the surgical technique has been described step by step through means of cadaveric dissection and then, the retrospective analysis of 385 patients affected by drug-resistant mesial temporal lobe epilepsy treated with this original subpial technique is reported. The results show that in a long-term follow-up, nearly 84% of the patients were free from seizures at the last follow-up, without permanent neurological deficits. According to the findings of this study, the anterior sylvian point represents a safe cortical landmark useful in mesial temporal lobe surgery because constantly present and anterior to risky temporal regions such as temporal horn and language networks.

In the last article of this Research Topic, the focus is moved to purely anatomical study based on neuroimaging. Baena-Caldas et al. in their article entitled “*Anatomical Variations of the Atlas Arches: Prevalence Assessment, Systematic Review, and Proposition for an Updated Classification System*” report a reviewed classification of atlas arches anatomical variants. In this article, the Authors applied Currarino's classification to a wide population of 839 patients and they analyzed the craniovertebral junction anatomy utilizing a Cone-Beam Computed Tomography. Moreover, they performed a systemic review of the Literature and from the results of their study they propose a novel classification based on Currarino's. This classification could be helpful in diagnosis, surgical planning, preventing iatrogenic incidents, and designing rehabilitation strategies.

As Guest Editors of this Research Topic, we believe that the readership of “Frontiers in Medicine” will find interesting and novel information regarding the impact of neuroanatomy on modern research and clinical practice.

